# Neuropsychological and Neuropsychiatric Features of Chronic Migraine Patients during the Interictal Phase

**DOI:** 10.3390/jcm12020523

**Published:** 2023-01-09

**Authors:** Elena Lozano-Soto, Álvaro Javier Cruz-Gómez, Raúl Rashid-López, Florencia Sanmartino, Raúl Espinosa-Rosso, Lucía Forero, Javier J. González-Rosa

**Affiliations:** 1 Department of Psychology, University of Cadiz, 11003 Cadiz, Spain; 2 Institute of Research and Biomedical Innovation of Cadiz (INiBICA), 11009 Cadiz, Spain; 3 Department of Neurology, Puerta del Mar Universitary Hospital, 11009 Cadiz, Spain; 4 Department of Neurology, Jerez de la Frontera University Hospital, 11407 Jerez de la Frontera, Spain

**Keywords:** chronic migraine, interictal phase, neuropsychiatry, neuropsychological impairment

## Abstract

This study aimed to examine the presence of neuropsychological deficits and their relationships with clinical, pharmacological, and neuropsychiatric characteristics in chronic migraine (CM) patients assessed during a headache-free period. We enrolled 39 CM patients (mean age: 45.4 years; male/female ratio: 3/36) and 20 age-, sex-, and education-matched healthy controls (HCs, mean age: 45.5 years; male/female ratio: 2/18) in a case–control study. All CM patients underwent a full and extensive clinical, neuropsychiatric, and neuropsychological evaluation to evaluate cognitive domains, including sustained attention (SA), information processing speed (IPS), visuospatial episodic memory, working memory (WM), and verbal fluency (VF), as well as depressive and anxiety symptoms. CM patients exhibited higher scores than HCs for all clinical and neuropsychiatric measures, but no differences were found in personality characteristics. Although more than half of the CM patients (54%) showed mild-to-severe neuropsychological impairment (NI), with the most frequent impairments occurring in short- and long-term verbal episodic memory and inhibitory control (in approximately 90% of these patients), almost half of the patients (46%) showed no NI. Moreover, the severity of NI was positively associated with the number of pharmacological treatments received. Remarkably, disease-related symptom severity and headache-related disability explained global neuropsychological performance in CM patients. The presence of cognitive and neuropsychiatric dysfunction during the interictal phase occurred in more than half of CM patients, increasing migraine-related disability and possibly exerting a negative impact on health-related quality of life and treatment adherence.

## 1. Introduction

Migraine is one of the most common primary headache disorders, affecting 14% of the general population [1] and deleteriously impacting patients, families, and the health care system [2,3]. Recently, migraines have been identified as the second leading cause of disability worldwide among all ages and genders [4].

Migraine is mainly characterized by recurrent attacks of throbbing unilateral headaches that usually worsen with physical activity and are associated with symptoms such as nausea and sensitivity to sensory stimulation [5]. There is high variability in the intensity, duration, and frequency of headache attacks among migraine patients. According to attack frequency, migraine can be divided into two major clinical subtypes: episodic migraine (EM), in which patients experience headache attacks on fewer than 15 days per month, and chronic migraine (CM), in which patients experience headache attacks at least 15 days per month for >3 months [6].

Headache attack frequency and chronification have been linked to an increased risk of worse clinical outcomes [7]. Several studies have revealed that compared to EM patients, CM patients exhibit higher disability levels, reduced quality of life, higher rates of psychiatric comorbidities [8,9,10,11], and complications related to medication overuse [12]. Although the comorbidity of psychiatric disorders with migraine has been widely reported in previous studies [8,13], some authors have specifically suggested that CM patients exhibit more severe depression and anxiety symptomatology than EM patients [14,15,16]. More recently, CM patients have been found to exhibit significantly worse neuropsychological performance than EM patients [17,18], highlighting migraine chronification as a risk factor for cognitive decline. Additionally, several studies have suggested that higher levels of anxiety and depression may negatively influence the cognitive performance of CM patients [2,19], although the causal relationship between these variables is not yet fully understood.

Furthermore, migraine patients frequently report cognitive complaints that persist beyond acute migraine attacks, mainly in terms of attention and memory, which also contribute to migraine attack-related disability [2,20,21]. These impairments have promoted greater interest in neuropsychological impairment (NI) in this population. Although it is widely accepted that NI is common during the premonitory and headache phases of a migraine attack [21,22], there is less consensus concerning its presence during headache-free or interictal periods. While some studies have reported worse cognitive performance of migraine patients during the interictal phase than of healthy controls (HCs), [17,23,24,25] other studies have not found significant differences between the groups [26,27,28].

It seems reasonable that methodological differences between studies could explain, at least in part, these mixed results. Indeed, differences in sample recruitment methods (i.e., population vs. clinic-based), presence of psychiatric comorbidities (e.g., depression and anxiety), clinical characteristics of patients (e.g., headache attack frequency and thus differential diagnosis between EM and CM), and heterogeneity in the pharmacological treatments received have previously been demonstrated to influence cognitive performance [18,21,29,30]. However, these factors are not usually fully accounted for in most of the studies addressing NI in migraine. In addition, the lack of a specific neuropsychological battery for migraine patients has led to wide variability in the neuropsychological tests employed, which limits the ability to compare results among studies.

Despite growing interest in CM, it is difficult to specify the common neuropsychiatric and neuropsychological findings across migraine patients, as several investigations, particularly those investigating CM, have mainly screened for cognitive function during the aura or headache attack phase. As there is no clear consensus about the persistence of NI during the interictal phase [21,25,31], the aim of the present study was to explore the presence, prevalence, and severity of NI during the interictal phase (i.e., between migraine attacks; the headache-free period) in a homogenous cohort of CM patients and to determine the relationships of NI with clinical features, personality traits, neuropsychiatric status, and pharmacological treatments. We hypothesized that CM patients manifest variations in NI, including a constellation of alterations in different cognitive domains accompanied by the presence of significant mood symptoms during the interictal period; these variations may be associated with the number of failed treatments and the impact of headache-related disability.

## 2. Materials and Methods

### 2.1. Participants

A total of 59 participants (mean age: 45.4 years; male/female ratio: 5/54) were enrolled in this cross-sectional study: 39 (mean age: 45.4 years; male/female ratio: 3/36) patients with clinically defined CM without aura from the Cephalea Unit of the Neurologic Department at the Puerta del Mar University Hospital in Cadiz (Spain) and 20 HCs (mean age: 45.5 years; male/female ratio: 2/18) matched for sex, age and educational attainment with no diagnosis of migraine or neurological or psychiatric disorders. Recruitment and selection of included participants were performed by a clinical neurologist who verified the study criteria and carried out a standard clinical evaluation, including collection of medical history and current treatments, as well as a physical examination.

The inclusion criteria were as follows: (i) diagnosis of CM according to the International Classification of Headache Disorders (ICHD)-3 criteria [5]; (ii) no other ICHD-3, neurological, or psychiatric diagnosis; (iii) aged between 18 and 60 years; and (iv) a headache-free period lasting at least 4 days before the assessment to avoid possible transient effects on cognitive performance.

All selected participants provided written informed consent to participate in the study, which was approved by the Cadiz Biomedical Research Ethics Committee (Ref.: RER-TOX-2018-01).

### 2.2. Measures

Eligible study participants underwent a neurological examination and an extensive neuropsychological assessment to obtain data regarding their clinical, cognitive, and psychiatric status.

### 2.3. Clinical Data

The clinical assessment included the following:

- Visual analog scale (VAS) [32] to determine subjective headache severity both at the moment of the evaluation and during the last migraine attack;
- Headache Impact Test (HIT-6) [33] to provide a global measure of adverse headache impact;
- Migraine Disability Assessment Scale (MIDAS) [34] to assess disability levels.

To explore possible associations with clinical and neuropsychological variables, data regarding current pharmacological treatments taken by CM patients were also collected based on the following classifications [35]:

- Preventive therapies, including medications such as antiepileptics, beta-blockers, calcium channel blockers, antidepressants, and botulinum toxin type A (OnabotA).
- Abortive treatments include simple pain relievers, nonsteroidal anti-inflammatory drugs (NSAIDs), and triptans.

### 2.4. Neuropsychological and Neuropsychiatric Assessments

The neuropsychological evaluation included the Matrix Reasoning subtest from the Wechsler Adult Intelligence Scale, third edition (WAIS-III) [36], to obtain an intelligence quotient (IQ) index. The following 8 tests (resulting in a total of 17 scores) were administered to evaluate the respective cognitive functions:

- *Symbol Digit Modalities Test* (SDMT) [37]: information processing speed (IPS) and sustained attention (SA);
- *Selective Reminding Test* (SRT): learning and long-term verbal episodic memory, including long-term storage (LTS), consistent long-term retrieval (CLTR), and delayed recall (DR);
- *10/36 Spatial Recall Test* (SPART) [38]: learning and long-term visuospatial episodic memory, including immediate recall (IR) and DR;
- *Digit Span subtest* from the WAIS-III [36]: including both *digit span forward* (DF) to assess short-term memory (STM) and *digit span backward* (DB) to assess working memory (WM);
- Verbal fluency tests (the PMR test [39]) and semantic/category fluency (the Animal Naming test [40]): phonemic verbal fluency (PVF), semantic verbal fluency (SVF), and executive function (EF);
- *Trail Making Test* (TMT; parts A and B) [41]: IPS and EF, including planning, inhibitory processing, and decision making;
- *Tower of London* (TOL) [42]: WM, prospective memory, SA, and control of interference, including four subscores (number of problems correctly solved, number of total moves performed, total time required, and number of rule violations);
- computerized version of the *Stroop color-word test* [43]: SA, IPS, inhibitory control, and resistance to interference (see Supplementary Materials for further description).

Psychiatric symptomatology was assessed with the following questionnaires:

- The Beck Depression Inventory-II (BDI-II) [44];
- State-Trait Anxiety Inventory (STAI) [45].

Finally, personality traits were assessed with the NEO Five Factor Inventory (NEO-FFI) [46] including specific scores on neuroticism (*n*), extraversion (E), openness to experience (O), agreeableness (A), and conscientiousness (C). The possible contributions of these personality traits to clinical and cognitive variables were also explored.

### 2.5. Procedures

#### 2.5.1. Neuropsychological Impairment Definition and Global Cognitive Z Score Calculation

In CM patients, failure on each neuropsychological test was based on raw scores falling more than 1.5 standard deviations (SDs) below the mean values of the HC group [47]. Unlike the criteria of 1 or 2 SDs below normative scores, which may overestimate or underestimate the presence of NI, respectively, adopting a −1.5 SD cutoff criterion has been suggested as the optimal threshold for detecting abnormal performance on a neuropsychological test [48,49].

The absence of NI was defined as failing 3 or fewer neuropsychological tests (20% of tests) [50]. NI severity was determined based on the number of failed tests as mild (4–7 failed tests), moderate (8–10 failed tests), or severe (more than 11 failed tests).

In addition, Z scores for each of the seventeen neuropsychological raw subscores were obtained using the mean and SD of the HC group (Z score = [Raw score – HC mean score]/HC SD). In the next step, a global cognitive Z (ZG) score was obtained for each participant, which represented the average Z score across all the administered tests.

#### 2.5.2. Statistical Analysis

All statistical analyses were conducted with SPSS 24.0 (IBM Corp., Armonk, NY, USA) on an anonymized database. The Shapiro–Wilk test was used to verify the normal distribution of all continuous variables. Among all variables collected, SRT-CLTR, SPART-IR, SDMT, PVF, SVF, Stroop interference, MIDAS, STAI-S, STAI-T, and NEO-PI (E, O, A, and C) scores showed a normal distribution, whereas SRT-LTS, SRT-DR, SPART-DR, DF, DB, TMT-A and B, TOL (4 subscores), Stroop errors, VAS (evaluation and attack), HIT-6, BDI-II and NEO-PI (*n*) scores were not normally distributed.

Student’s t tests and Mann–Whitney U tests, as appropriate, were used to examine differences between the CM and HC groups. Chi-square (χ^2^) tests were used to assess differences between groups for dichotomized variables (sex) and the classification of pharmacological treatments.

Pearson and Spearman correlation analyses were performed to determine the association between the identified significant results. In addition, chi-square tests were used to determine whether NI severity was distributed proportionally across the number of pharmacological treatments received.

Multiple linear regression analyses using a forward selection procedure were then conducted in the CM group to assess whether clinical and neuropsychiatric measures were associated with overall cognitive status (i.e., ZG score). 

## 3. Results

Participant demographic characteristics are summarized in Table 1. The CM and HC groups did not significantly differ based on sex (*p* = 0.768), age (*p* = 0.923), or educational attainment (*p* = 0.506). Both the CM and HC groups were predominantly female (92.3% and 90%, respectively), with similar mean ages (45 years) and educational attainment (mainly between lower and upper secondary education; ISCED 2011).

Specific information concerning current medication use by CM patients at the time of the evaluation is also presented in Table 1. Nearly 76.9% of the patients were currently taking two or more medications, and only 5.1% of the patients were not taking medications.

Clinical data obtained through specific questionnaires assessing headache-related pain, impact, and disability are also provided in Table 1. As expected, CM patients showed higher scores than HCs in subjective reports of headache pain intensity (VAS scores) both during the evaluation (*p* < 0.001) and the last migraine attack (*p* < 0.001), migraine-related disability levels (MIDAS scores, *p* < 0.001), and impact on daily functioning (HIT-6 scores, *p* < 0.001). Furthermore, CM patients exhibited worse results than HCs on neuropsychiatric scales, indicating more severe depressive symptoms (BDI-II scores, *p* < 0.001) and greater trait anxiety (STAI-T scores, *p* < 0.001); no significant differences were found regarding personality traits assessed through the NEO-FFI (see Table 1).

**Figure 1:**
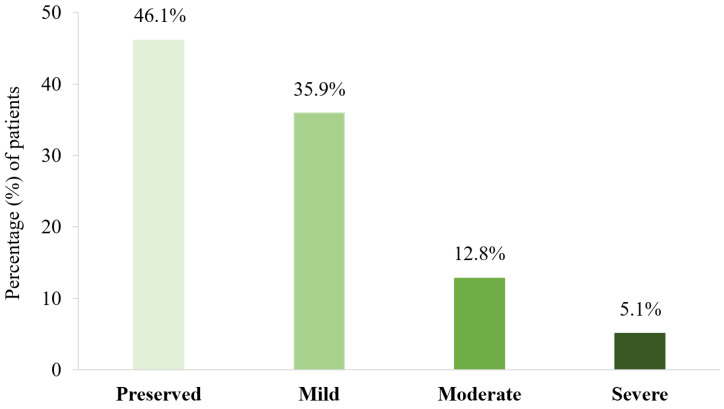
Percentages of CM patients displaying different degrees of neuropsychological impairment. Preserved: failed 3 or fewer neuropsychological tests; Mild: failed 4–7 neuropsychological tests; Moderate: failed 8–10 neuropsychological tests; Severe: failed 11 or more neuropsychological tests. Impaired neuropsychological performance (i.e., test failure) was defined as scoring < 1.5 SD below the normative values.

Regarding the participants’ cognitive status, the neuropsychological scores of the CM patients and HCs are presented in Table 2. There were significant differences between the groups in SRT (LTS, CLTR, and DR scores), DF, and Stroop interference scores, indicating worse performance of CM patients than HCs. No significant group differences were found in the remaining neuropsychological tests. Notably, after applying normative corrections, 46.1% of patients were classified as having no NI (i.e., neuropsychologically preserved), while 53.9% of patients exhibited NI (failed three or more neuropsychological tests). Among CM patients with NI, 35.9% were classified as having mild NI, 12.8% were classified as having moderate NI, and 5.1% were classified as having severe NI (Figure 1). Specifically, the Stroop interference index and SRT (CLTR and DR scores) were the most common cognitive tests on which CM patients exhibited impaired performance (41% and 35.9%, respectively), followed by the SRT-LTS (33.3%) and SVF scores (30.7%). Remarkably, 90% of CM patients with NI showed abnormal performance on the SRT (CLTR and DR subscores), Stroop test (interference subscore), or both tests.

Although no significant relationships were found between pharmacological treatments and neuropsychological variables, an additional exploratory analysis revealed that more than half (54%) of CM patients with mild, moderate, or severe NI took two or more than two medications. Thus, the severity of NI was associated with the number of treatments received (χ^2^ = 23.1; *p* = 0.027).

**Figure 2:**
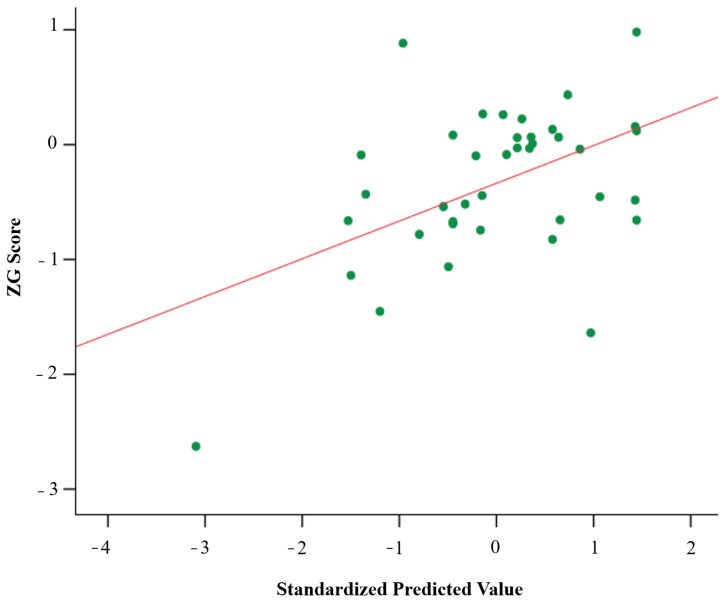
Associations between standardized predicted values and observed global neuropsychological scores. Scatterplot with a trend line depicting the correlations between global cognitive (ZG) scores predicted from multiple regression analyses, with ZG scores observed as the dependent variable and MIDAS (β = –0.348) and VAS (attack) scores (β = 0.409) as the independent variables.

Finally, multiple regression analysis was performed, including ZG scores as the dependent variable and VAS, MIDAS, HIT-6, BDI-II, STAI, and NEO-FFI as independent variables. The VAS (attack; b = 0.409; t = 2.76, *p* = 0.009) and MIDAS scores (b = − 0.348; t = −2.35, *p* = 0.024) were the variables retained in the final model (R^2^ = 0.24; *p* = 0.008) and predicted global neuropsychological performance. This finding showed that lower disability predicted higher global neuropsychological performance in CM patients (Figure 2).

## 4. Discussion

The main aim of our study was to investigate the presence of NI in a well-characterized homogenous sample of CM patients during the interictal period and to explore the association of CM with clinical and neuropsychiatric outcomes. Our research revealed that CM patients exhibited variable NI during periods between acute migraine attacks. In particular, the patients exhibited cognitive impairment in SA, verbal episodic memory, and Stroop-like interference. Our results also revealed that these cognitive abnormalities were not explained by personality traits and were present in approximately half of the patients. Moreover, these impairments may be partially due to disease-related symptom severity and disability, cumulative history of medication use for acute treatment, and the treatment response of the patients.

As we mentioned before, there is a lack of consensus in the literature regarding the presence of NI in migraines. While some studies have not reported worse cognitive performance in migraine patients than in HCs [26,27,51,52], others have reported lower neuropsychological performance in migraine patients [17,18,23,24,29,53,54,55]. These inconsistent results may be partially explained by the lack of specific CM diagnostic criteria, clarification of whether patients were evaluated during the ictal or interictal period, sample size specifications, or an adequate control group. Furthermore, to date, there is no consensus about what cognitive domains should be evaluated; therefore, studies have exhibited substantial variation in the neuropsychological tests administered and in the cutoff points used to classify cognitive impairment, potentially resulting in a lack of homogeneity.

To address this issue, we used an extensive neuropsychological battery (composed of 8 tests including 17 subscores) to assess cognitive domains not always included in previous studies that included only a few neuropsychological tests or screening tests, such as the Montreal Cognitive Assessment (MOCA) or Mini-Mental State Examination (MMSE) [29,54]. In addition, we recruited a well-defined, homogeneous group of individuals who were diagnosed with CM by expert neurologists (following the ICHD-3 criteria), whereas in some population-based studies, diagnostic accuracy may have been influenced by self-reports [52,56,57]. Moreover, our study included a homogenous cohort comprised exclusively of CM patients; previous studies included migraine patients without clinically distinguishing between EM and CM and thus did not control for migraine attack frequency [26,29,51,56]. Thus, several factors bolstered the reliability of our results, which indicated the presence of NI in CM patients. Specifically, we used a comprehensive neuropsychological battery and clinic-based recruitment and ensured the clinical homogeneity of the cohort of CM patients who were assessed only during the interictal period.

The mechanism underlying interictal NI remains elusive, but numerous neuroimaging studies have consistently shown the presence of extensive structural and functional brain abnormalities involving both cortical and subcortical regions important for cognitive processing that occur during attacks [58,59,60]. Additionally, seminal studies have revealed that even in the interictal period, the brains of migraine patients process sensory information differently [61,62], which may explain why many of these patients show cognitive disturbances during the interictal phase.

The severity of NI has been directly related to migraine severity characteristics such as disease duration, frequency, and intensity of headache attacks [21,63] Accordingly, some studies have reported significantly lower cognitive performance of CM patients compared to that of EM patients, who by definition have less frequent headache attacks [17,18]. Consistent with our results, other studies involving CM patients have also demonstrated the presence of NI with impacts on several cognitive domains, including attention [24], short-term memory [24], episodic memory [17,53], verbal fluency [24], executive function [18,53] and inhibitory control [24]. Remarkably, our results were not explained by demographic differences since we matched CM patients with HCs in terms of age, sex, and educational attainment. In addition, to control elements that could interfere with our results, we ensured that CM patients were assessed only during the interictal period. To this end, we used two VAS evaluations to compare the headache experienced during an evaluation and the headache experienced during an attack.

In addition to cognitive impairment, CM also causes a varying extent of disability, and both the frequency and intensity of headache attacks most influence patient quality of life [64]. Similarly, according to the MIDAS and VAS (attack) results, our patient group presented higher scores in disability and frequency of headache than the HC group, which indicates severe migraine parameters and may be partially responsible for the profile of cognitive disturbances exhibited by CM patients, even during the interictal phase.

According to the literature, migraine is associated with a high disability burden [4], affects patient quality of life, and therefore, causes a strong emotional impact that may lead to the development of psychiatric disorders in this population. Specifically, there is evidence that CM patients suffer more psychiatric symptoms than EM patients [14,15]. Although a bidirectional relationship between disability levels and psychiatric symptoms in migraine patients has been proposed [15], other authors have suggested that psychiatric comorbidities may represent a risk factor for migraine chronification [65]. Consistent with this finding, our CM patients exhibited higher scores on the BDI-II and STAI than HCs.

Moreover, MIDAS scores were closely related to BDI-II scores in our CM cohort, demonstrating that headache-related disability is influenced by the severity of depressive symptoms, which may constitute the psychopathological profile of patients [66,67]. These results support the idea that psychiatric comorbidities, such as depressive problems, in migraine are frequent and disabling [15] and should be seriously considered in clinical management.

Some limitations of this study should be noted. First, due to the large variety of treatments that the patients received, it was not possible to stratify these treatments for analysis; however, treatment could influence cognitive test performance, as discussed. Numerous studies have reported this same problem [21,25]. Second, although several authors have suggested that the severity of cognitive abnormalities in CM may be related to neuropathological findings from neuroimaging and neurophysiological studies [68,69,70,71], the lack of a brain imaging component in our study prevented us from assessing structural and functional brain alterations and their contributions to NI. Future studies should include structural or functional imaging data to better document brain changes related to basic cognitive processes in CM. Third, the sample size was relatively small; therefore, the current findings should be interpreted with caution since they may not necessarily reflect the cognitive function of community-dwelling patients or individuals suffering from milder forms of migraine. Nevertheless, a major strength of this study is the strict inclusion criterion for patients with CM based on a well-established clinical diagnosis. Moreover, patients were specifically assessed between attacks (in the absence of headache symptoms) during a time in which patients’ cognitive function was supposed to function normally with no persisting symptoms, which decreased the possibility that migraine itself affected test performance and increased the accuracy of the study. Finally, due to the low number of male participants with respect to female participants (in both the CM and HC groups), findings regarding male CM patients need to be interpreted cautiously.

## 5. Conclusions

Our results provide evidence that CM can be accompanied by a variety of cognitive symptoms during the interictal phase. These neuropsychological symptoms occurred in half of the CM patients evaluated, were specific to tests targeting short- and long-term verbal memory, SA, and resistance to interference, and were unrelated to migraine attacks. Importantly, these cognitive impairments are most likely related to the mechanisms underlying migraine-induced disability, as they were more frequent in patients who experienced failure of previous treatments and were not related to other comorbidities, such as mood disorders or personality traits. The impacts of migraine severity and migraine-related disability can thus be considered the strongest cause of interictal cognitive impairment in many CM patients.

## Figures and Tables

**Table 1:** Demographic, clinical, and neuropsychiatric characteristics of CM patients.

	CM (*n* = 39)	HCs (*n* = 20)	*t*/U/χ^2^	*p* Values
*Demographic characteristics*				
Age	45.4 (10.4)	45.5 (9.6)	0.096	0.923
Education (years)	10.9 (3.0)	11.7 (3.3)	−0.666	0.506
Sex	3 M/36 F	2 M/18 F	0.763	0.768
*Clinical characteristics*				
VAS (evaluation)	3.2 (2.1)	0.7 (1.6)	−4.54	**0.001**
VAS (attack)	9.7 (0.6)	6.2 (2.7)	−5.40	**0.001**
MIDAS	65.5 (45.5)	3.9 (10.2)	8.06	**0.001**
HIT-6	68.3 (7.0)	44.4 (12.0)	−5.67	**0.001**
*CM patients: Current treatment*				
Preventive therapy				
Antiepileptics	6 (15.4%)			
Beta-blockers	3 (7.7%)			
Calcium channel blockers	1 (2.6%)			
Antidepressants	11 (28.2%)			
TBX	18 (46.1%)			
Abortive therapy				
Simple pain relievers	6 (15.4%)			
NSAIDs	18 (46.1%)			
Triptans	19 (48.7%)			
Benzodiazepines	4 (10.2%)			
*Neuropsychiatric screening scores*				
BDI-II	19.9 (10.3)	6.5 (5.8)	−5.01	**0.001**
STAI-S	25.0 (12.0)	18.2 (13.6)	1.95	0.056 ^§^
STAI-T	30.6 (12.1)	19.7 (7.1)	4.36	**0.001**
NEO-FFI (*n*)	34.6 (5.9)	32.4 (4.6)	−1.42	0.157
NEO-FFI (E)	35.8 (3.7)	34.0 (4.1)	−1.70	0.095
NEO-FFI (O)	37.6 (3.6)	39.0 (4.1)	−1.37	0.175
NEO-FFI (A)	38.7 (4.3)	38.4 (2.8)	−0.20	0.839
NEO-FFI (C)	38.2 (3.0)	39.6 (3.2)	−1.67	0.101

Means and standard deviations (SD) for each group are presented for continuous variables; frequency values and percentages are presented for categorical variables (sex and medication). BDI-II: Beck Depression Inventory-II; CM: chronic migraine; HCs: healthy controls; HIT-6: Headache Impact Test; MIDAS: Migraine Disability Assessment Scale; NEO-FFI: NEO Five Factor Inventory; (A): Agreeableness; (C): Conscientiousness; (E): Extraversion; (N): Neuroticism; (O): Openness to experience; NSAIDs: nonsteroidal anti-inflammatory drugs; STAI-S: State-Trait Anxiety Inventory—State score; STAI-T: State-Trait Anxiety Inventory—Trait score; VAS: Visual Analog Scale. Note: § = statistical trend; bold values indicate significant differences, *p* > 0.05.

**Table 2:** Neuropsychological data of CM patients and HCs.

*Neuropsychological Tests*	CM (*n* = 39)	HCs (*n* = 20)	*t*/U	*p*-Values
IQ (WAIS-III Matrix Reasoning subtest)	100.5 (12.5)	105.7 (15.8)	−1.23	0.219
SRT-LTS	46.7 (12.8)	52.6 (9.2)	−2.01	**0.044**
SRT-CLTR	35.6 (14.1)	46.2 (12.1)	−2.89	**0.005**
SRT-DR	8.8 (2.4)	10.2 (1.8)	−2.04	**0.042**
SPART-IR	17.5 (5.1)	18.6 (6.0)	0.75	0.456
SPART-DR	6.0 (2.5)	7.0 (2.3)	−1.67	0.095
SDMT	50.0 (11.6)	54.9 (11.3)	−1.56	0.124
DF (WAIS-III)	5.2 (1.0)	6.1 (1.0)	−2.52	**0.012**
DB (WAIS-III)	4.4 (1.3)	4.6 (1.4)	−0.74	0.462
TMT-A (Time)	30.6 (11.3)	32.0 (10.0)	−0.67	0.501
TMT-B (Time)	75.5 (47.2)	65.5 (28.8)	−0.88	0.378
Phonemic Fluency (PMR)	37.4 (14.1)	40.9 (9.2)	−0.99	0.325
Semantic Fluency (Animals)	19.5 (5.6)	21.7 (3.3)	−1.89	0.064
TOL (Total Correct)	9.1 (1.3)	8.7 (1.6)	−0.65	0.517
TOL (Total Moves)	70.2 (15.4)	73.2 (13.1)	−0.73	0.466
TOL (Total Time)	197.8 (79.1)	197.5 (47.9)	−1.17	0.242
TOL (Rule Violations)	1.1 (1.5)	1.2 (1.0)	−1.45	0.135
Stroop (Error)	24.0 (23.4)	23.0 (23.2)	−0.36	0.718
Stroop (Interference)	64.0 (34.2)	49.0 (16.9)	2.23	**0.028**

Raw score means and standard deviations (SD) are presented for each test. CM: chronic migraine; CLTS: consistent long-term retrieval; DB: digit span backward; DF: digit span forward; DR: Delayed recall; HCs: healthy controls; IR: immediate recall; IQ: intelligence quotient; LTS: long-term storage; TMT-A: Trail Making Test Part A; TMT-B: Trail Making Test Part B; TOL: Tower of London; SDMT: Symbol Digit Modalities; SPART: 10/36 Spatial Recall Test; SRT: Selective Reminding Test; WAIS: Wechsler Adult Intelligence Scale. Note: bold numbers indicate significant differences (*p* < 0.05).

## Data Availability

The datasets generated and analyzed in the current study are available from the corresponding author upon reasonable request.

## Supplementary Materials

The following supporting information can be downloaded at: https://www.mdpi.com/article/10.3390/jcm12020523/s1, Figure S1: Computerized Stroop task and trial structure.
