# Supplementary material for: Neuropsychological and Neuropsychiatric Features of Chronic Migraine Patients during the Interictal Phase
Source: J Clin Med. 2023 Jan 9;12(2):523. doi: 10.3390/jcm12020523 (PMC9864628; doi:10.3390/jcm12020523)
Supplement: Supplementary file 1 [file jcm-12-00523-s001.zip › jcm-2030467-supplementary.pdf]

Supplemental Material

A computerized version of the Stroop test (Mead et al., 2002) was administered individually to each participant, taking approximately 5 minutes, which was designed andadministered using E-Prime 2.0 software (https://www.pstnet.com/). The task (Supplementary Figure S1) was composed of a total of 300 trials divided into two blocks (150 trials per block). In each block, three experimental conditions were randomly presented: congruent, incongruent and neutral (50 trials per experimental condition). Fourcolors and words written in Spanish ("rojo" [red], "azul" [blue], "amarillo" [yellow], and"verde" [green]) were displayed on a black background on a 17-inch monitor and randomly alternated in various combinations. The experimental conditions are specified as follows: i) *color-congruent trials* (congruent): four colors and words written were printed in the corresponding ink color; ii) *color-incongruent trials* (incongruent): the fourwords were randomly alternated in various combinations of colors; and iii) *color-neutraltrials* (neutral): for the design of this experimental condition, showing four animal nameswritten in Spanish ("mariposa" [butterfly], "vaca" [cow], "perro" [dog], and "gato" [cat])that were selected to match the number of letters in the colored words were presented using the four ink color possibilities.

The participants were seated in front of a screen placed approximately 100 cm from the eyes with their dominant hand positioned palm down on a homemade response pad. Responses were made by pushing one of four buttons, one for each of the four colors, which were mounted and randomly swapped between participants on the response box. Both the hands' index and middle fingers were used to press the buttons. The participantswere instructed to maintain their gaze on a fixation cross for 800 ms throughout the experiment and respond to stimuli as quickly and accurately as possible by pressing the colored button that matched the ink color of the presented words. The stimuli appeared for 400 ms, and the interstimulus interval (ISI) was 600–1000 ms.

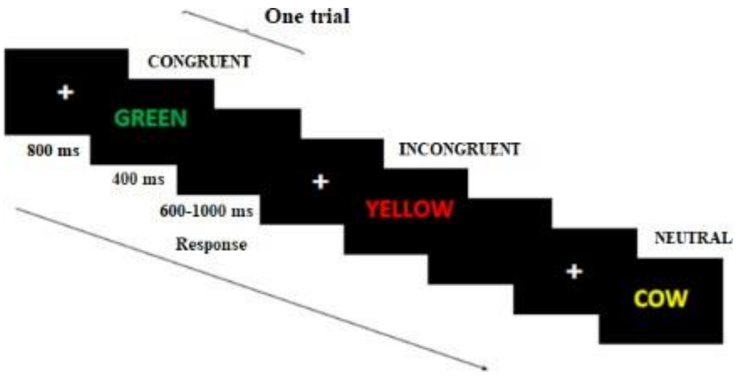

Supplementary Figure S1. Computerized Stroop task and trial structure
